# Effect of Pigmented Rice Consumption on Cardiometabolic Risk Factors: A Systematic Review of Randomized Controlled Trials

**DOI:** 10.1007/s13668-023-00496-7

**Published:** 2023-09-07

**Authors:** Diane Mendoza-Sarmiento, Emmanuele V. Mistades, Alison M. Hill

**Affiliations:** 1 Graduate School, University of Santo Tomas, Manila, Philippines; 2 Nutrition and Dietetics Department, College of Education, University of Santo Tomas, Manila, Philippines; 3 Clinical and Health Sciences, Alliance for Research in Exercise, Nutrition and Activity, University of South Australia, Adelaide, Australia

**Keywords:** Pigmented rice, Cholesterol, Glucose, Weight, Blood pressure

## Abstract

**Purpose of Review:**

Dietary patterns that include polyphenols may help manage cardiometabolic risk factors. Pigmented rice contains phenolic acids and flavonoids that contribute to its antioxidant properties. This review examined the effect of polyphenol-containing pigmented rice on antioxidant status, lipid profile, glucose/insulin, blood pressure, and weight among adults. Four electronic databases including PubMed, ProQuest, EBSCOhost, and Google Scholar were systematically searched for relevant articles published in English since 2000, using PRISMA guidelines (PROSPERO registration: CRD42022358132). Two-staged screening resulted in the inclusion of seventeen (seven acute, ten chronic) randomized controlled trials. A random effects model was conducted on cardiometabolic outcomes reported in at least three studies.

**Recent Findings:**

Acute intake increased plasma antioxidant activity and lowered postprandial glucose and insulin levels. Chronic consumption was associated with reductions in fasting glucose (WMD: -1.60 mg/dL; 95% CI:-3.05,-0.14, *p* = 0.03*, k* = 5, *n* = 349), weight (WMD: -0.23 kg, 95% CI: -0.44, -0.02, *p* = 0.03,* k* = 3, *n* = 182), and diastolic blood pressure (WMD: -1.39 mmHg, 95% CI: -2.21, -0.56, *p* = 0.001, *k* = 3, *n* = 185). No effect on total cholesterol, low-density lipoprotein, high-density lipoprotein, triglycerides, body mass index, and systolic blood pressure was found.

**Summary:**

The consumption of pigmented rice may improve cardiometabolic risk factors. However, the small number of studies and differences in study design, including participants’ health status, form of rice utilized, and duration of intervention, support the need for more high-quality trials to further investigate these findings.

**Supplementary Information:**

The online version contains supplementary material available at 10.1007/s13668-023-00496-7.

## Introduction

Cardiometabolic risk factors such as dyslipidemia [1, 2], overweight and obesity [2, 3], hyperglycemia [1], and hypertension [1, 2, 4] influence the development of cardiovascular diseases (CVDs). Dyslipidemia, primarily elevated low-density lipoprotein (LDL) cholesterol, is recognized as an independent risk factor for developing CVDs [5], as when oxidized, induces regulatory T-cell apoptosis which promotes atherosclerosis [6]. Hyperglycemia promotes the formation of advanced glycation end products, which contribute to endothelial dysfunction [7], while obesity contributes to increased inflammation, insulin resistance, and dyslipidemia [8]. Elevated blood pressure (BP) increases the risk of vascular and cardiac damage [4].

Healthy diets that include polyphenols are shown to reduce cardiometabolic risk factors for CVDs [9]. Polyphenols are natural bioactive compounds synthesized by plants which contributes to their sensory and nutritional properties [10]. Polyphenols influence the development of CVDs by modulating inflammation, reducing LDL oxidation, and neutralizing free radicals [11–13]. Sources of polyphenols may vary depending on cultural and geographical factors [14]. Common sources are coffee and tea [15, 16], red wine [16, 17], fruits and vegetables, and cereals [15–18]. Rice *(Oryza Sativa L),* considered a staple in many countries [19•], contains several bioactive compounds, including polyphenols [20]. Specifically, pigmented rice which has colored bran fractions such as black, purple, and red [20], contains phenolic compounds, mainly ferulic acid, and flavonoids such as anthocyanins which influence its diverse colors [19•, 20]. Total anthocyanin content (TAC) and total phenolic content (TPC) are postulated to be correlated with antioxidant potential [21, 22]. Anthocyanins stabilize free radicals by donating an electron or hydrogen atom [23], and phenolic acids scavenge free radicals through hydrogen donation [24].

Animal models have identified several mechanisms through which pigmented rice may reduce cardiometabolic risk including the regulation of enzymes involved in metabolic pathways that influence lipid and glucose metabolism. Lower total cholesterol (TC), LDL, and triglycerides (TG) were attributed to a reduction in the expression of fatty acid synthase (FAS) [25, 26], an enzyme that catalyzes de novo synthesis of fatty acids, and an increase in carnitoyl transferase (CPT), which is essential for fatty acid oxidation [27]. Also demonstrated was increased levels of adiponectin [26], which is associated with improved insulin sensitivity, and reduced TG via increasing lipoprotein lipase activity [28]. Lastly, reduced adipose tissue and improved lipid levels were associated with down-regulation of peroxisome proliferator activated receptor—γ (PPAR-γ) [29]. Several human trials have recently been conducted to investigate the effect of pigmented rice consumption on lipids, glucose and body weight. However, the overall effect of pigmented rice on cardiometabolic risk factors has yet to be summarized. Thus, this review aimed to systematically review the body of evidence on the effect of pigmented rice on antioxidant status, cholesterol, glucose/insulin, BP, and weight in adults.

## Materials and Methods

This review was conducted according to PRISMA guidelines, and the protocol is registered in the PROSPERO database (CRD42022358132).

### Search Strategy

A core search strategy was first developed in PubMed by identifying MeSH terms for each concept (adult, pigmented rice, control, cholesterol, glucose, BP, phenolics, weight, and waist circumference (WC)), and using Boolean operators *(See* *supplementary information**)*. This was then used to systematically search for relevant studies in the following databases: PubMed, ProQuest, EBSCOhost, and Google Scholar (search completed October 25, 2022). In addition, online clinical trial registries (ClinicalTrials.gov, RIAT, Phil. Health Research Registry, and Australian New Zealand Clinical Trials Registry (ANZCTR)), reference lists of identified studies (pearling), and other sources of grey literature were searched to ensure all relevant studies were included. No limit on language was applied, but date of publication was limited from 2000 to the present as research outputs on the relationship between rice consumption and health increased from 2000 thereafter [20].

### Eligibility Criteria/Study Selection

Table 1 presents the summarized eligibility criteria used in the present study. Randomized-controlled trials with *(P)* adult participants (> 18 years old) using *(I)* pigmented rice (red, purple, and/or black) in any form (including cooked, extract, powdered) as an intervention against a *(C)* control (placebo, white rice, brown rice, maltodextrin, or usual diet), and reporting at least one of the following outcomes *(O)*: antioxidant status (total phenol index (TPI), total antioxidant/radical activity/capacity (TAC)), cholesterol (TC, LDL, high-density lipoprotein (HDL), TG), glucose/insulin (fasting blood glucose, glycated hemoglobin, insulin, insulin sensitivity), BP and anthropometry (weight, body mass index (BMI), WC/waist-hip ratio (W:H)) were included. Studies were excluded if they combined pigmented rice with other foods or intervention or did not compare to a control, such that the specific effects of pigmented rice could not be determined.

**Table 1:** PICOS criteria used to define the eligibility criteria

**Parameter**	**Description**
P-Population	Adults (> 18 years old)
I-Intervention/variable of interest	Pigmented rice (red, black, or purple)
C-Comparator	Control or placebo
O-Outcome	Antioxidant status, Cholesterol (TC, LDL, HDL, TG), Glucose/insulin, Blood pressure, Anthropometry
S-Study design	Randomized controlled trial

Studies were only eligible if published in full text in a peer-reviewed journal, in English (or with English translation). Animal and in vitro studies were excluded.

All studies were independently screened by title and abstract, then by full text by two reviewers (DMS; AMH, EVM, and RAR), and any disagreement was settled by a third reviewer (AMH, EVM, or RAR) using COVIDENCE software.

### Data Extraction

Two reviewers independently extracted data (DMS; EVM and FPP) using a pilot-tested template, checked for discrepancies, and resolved by consensus. Extracted data included the following: author’s name, year of publication, country where the study was conducted, study design, study duration, blinding, study population characteristics (sex, age, co-morbidities, ethnicity), intervention (number of participants assigned and completed, type of rice, form, dosage, polyphenol content), comparator (number of participants assigned and completed, type, form, dosage), and pre- and post-intervention values (mean and standard deviation (SD), *p*-value)) for antioxidant status, cholesterol, glucose/insulin, BP, and weight.

### Risk of Bias Assessment

The quality of included studies was assessed independently by two reviewers (DMS; EVM and RAR using the Cochrane Collaboration Risk of Bias Version 2 (RoB 2) tool, and a consensus resolved disagreements. Studies were assessed as low risk ( +), high risk (-) or some concerns (!), based on the following domains: (D1) bias arising from randomization process, (D2) bias due to deviations from intended interventions, (D3) bias due to missing outcome data, (D4) bias in measurement of outcome, and (D5) bias in selection of the reported result [30]. Crossover studies included assessment on bias arising from period carryover effects (DS).

### Data Analysis

Chronic and acute study data were assessed separately for each outcome. Antioxidant activity in acute studies were analyzed by identifying the time points for initial increase of antioxidant activity, peak response, and duration of response. Pre-and post-intervention mean and standard deviation (SD) for TC, LDL, HDL, TG, glucose, BP, weight, and BMI were used to compute weighted mean differences (WMD) with their 95% confidence interval, and overall effect size for chronic studies. Standard deviations were calculated for studies that reported only standard error of the mean (SEM) using the formula: SD = SEM x square root (*n*). Studies without reported change and/or SD were calculated using the following formulas assuming a modest correlation coefficient (*R*) of 0.5 [31].

$$\mathrm{Change}\; \mathrm{in}\; \mathrm{outcome}=\left(\mathrm{post}-\mathrm{treatment}-\mathrm{pre}-\mathrm{treatment}\right)$$

$${SD}_{change}=\sqrt{{SD}_{baseline}^{2}+{SD}_{final}^{2}-(2 \times R \times {SD}_{baseline}\times {SD}_{final} )}$$

A random effects model was conducted on cardiometabolic outcomes reported in at least three studies using Review Manager version 5.4 [32], with a *P-*value of < 0.05 considered statistically significant. Heterogeneity was assessed using chi-squared test and *I*^*2*^ statistics. An *I*^*2*^ result of > 50% was considered substantial heterogeneity [33]. Forest plots were produced to present the summarized information on each study, heterogeneity, and overall effect size for each outcome. Due to the low number of studies included in the analyses, funnel plots were not generated.

Quality of evidence for each outcome was rated high, moderate, low, or very low based on the following criteria: (1) risk of bias, (2) inconsistency of results, (3) indirectness of evidence, (4) imprecision, and (5) publication bias [34], using the Grading of Recommendations, Assessment, Development and Evaluations (GRADE) criteria [35].

## Results

### Study Selection

**Fig. 1:**
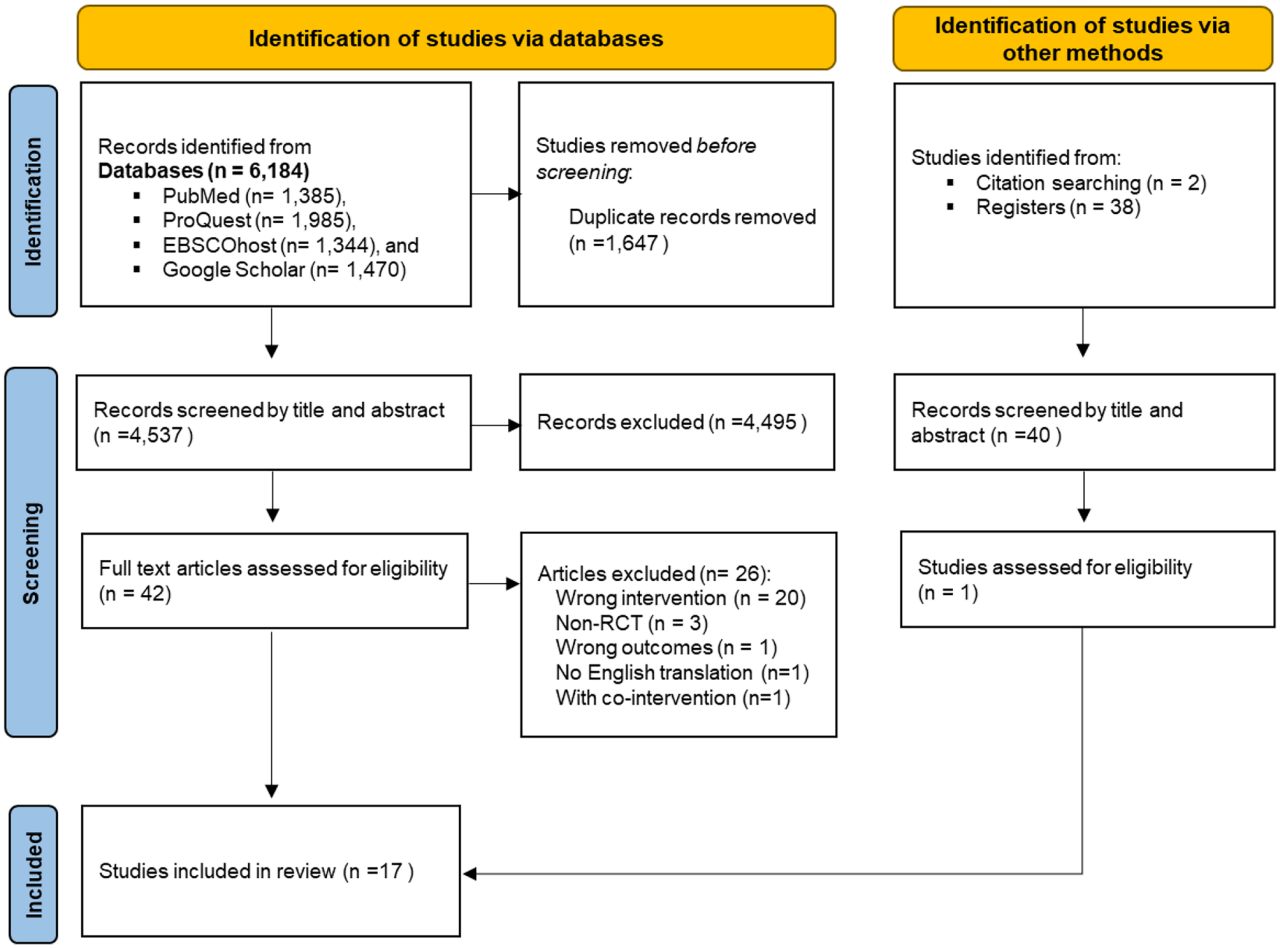
Flow diagram of study selection

Figure 1 presents the flow of study selection based on PRISMA guidelines [36]. A total of 6,184 studies from the four databases and 40 from additional searches were identified. Duplicates were removed using a reference manager (*Mendeley)*. A total of 4,537 studies were imported to COVIDENCE for screening. A total of 42 studies were identified for full-text review, of which 26 were excluded based on the following reasons: wrong intervention (n = 20), non-RCT (n = 3), wrong outcomes (n = 1), no English translation (n = 1), and with co-intervention (n = 1). Seventeen eligible studies were included in the systematic review.

### Study Characteristics

Characteristics of the included studies are presented in Tables 2 and 3. Of the 17 studies, seven were acute and ten were chronic interventions*.* Eight studies used a cross-over design [37–41, 42••, 43, 44], and nine studies were parallel [45, 46, 47•, 48–53].

**Table 2:** Acute study characteristics

**Reference**	**Country**	**Study Design**	**Number completed**	**Sex**	**Age (years) Mean ± SD**	**Health Status**	**Intervention**	**Polyphenol content**	**Control**	**Timepoints** **(minutes)**	**Outcomes**
Anuyahong et al. [38]	Thailand	Crossover	19	M,F	28.1 ± 3.0	Healthy	Purple Rice (Riceberry extract added to yogurt)	28.1 anthocyanin; 17.4 C3G; 7.9 P3G	Yogurt	30, 60, 90, 120, 150, 180	Postprandial plasma glucose, and antioxidant status (FRAP, TEAC, ORAC, MDA)
Anuyahong et al. [39]	Thailand	Crossover	13	M	24.46 ± 0.90	Overweight/obese	Purple Rice (Riceberry, extract)	67 mg gallic acid, C3G 2 mg g-1 0.8 mg P3G per gram extract	Sucrose	Glucose: 15,30, 60, 90, 120, 150, 180 Antioxidants:30, 60, 90, 120, 150, 180, 240, 300, 360	Postprandial plasma glucose, insulin - antioxidant status (FRAP, TEAC, ORAC, thiol)
Callcott et al. [40]	Australia	Crossover	22	M,F	47.57 ± 13.31	Obese	Red and Purple rice	NR	Brown rice	30, 60, 120, 360	Postprandial plasma antioxidant status (FRAP, MDA)
Chusak et al. [41]	Thailand	Crossover	16	M,F	24.29 ± 0.67	Healthy	Purple (Riceberry bread)	NR	Wheat bread	Glucose and Insulin: 15, 30, 60, 90, 120 Antioxidants: 30, 60, 90, 120, 150, 180	Postprandial plasma glucose, insulin - antioxidant status (FRAP, thiol, MDA)
Muangchan et al. [42••]	Thailand	Crossover	6	M	29 ± 5.14	Healthy	Purple (Riceberry)	NR	White rice	15, 30, 60, 120, 180	Postprandial plasma glucose and insulin
Se et al. [43]	Malaysia	Crossover	12	M,F	23.2 ± 1.4	Healthy	Red rice (UKMRC9, UKMRC10, UKMRC11, Thailand Red Rice)	TPC (%mg GAE/cooked rice): UKMRC (61.4 ± 2.59/178.9 g); UKMRC10 (81.7 ± 1.25/181.1 g); UKMRC 11 (55.2 ± 2.03/170.2 g); THAI RED (81.9 ± 3.53/174.2 g)	Basmati and jasmine	15, 30, 45, 60, 90, 120, 180	Postprandial plasma glucose and insulin
Vitalini et al. [44]	Italy	Crossover	19	M,F	24.7 ± 3.8	Healthy	Black rice (Venere and Artemide)	Venere (TP: 149 ± 13 mg gallic acid; TF: 240 ± 13 mg catechin; TA: 711 ± 76 mg) Artemide (TP: 806 ± 54 mg gallic acid; TF: 545 ± 31 mg catechin; TA: 614 ± 34 mg)	Brown	30, 60, 120, 180	Antioxidant activity (DPPH, ABTS)

*ABTS* 2,20-azino-bis(3-ethylbenzothiazoline-6-sulphonic acid, *C3G* cyanidin-3-glucoside, *DPPH* 2,2-diphenyl-1-picrylhydrazyl, *F* female, *FRAP* ferric reducing ability of plasma, *M* male, *MDA* Malondialdehyde, *NR* Not reported, *ORAC* oxygen radical absorbance capacity, *P3G* Peonidin-3-glucoside, *RCT* randomized controlled trial, *TA* Total Anthocyanins, *TEAC* Trolox equivalent antioxidant capacity, *TF* Total Flavonoids, *TP* Total polyphenols

**Table 3:** Chronic study characteristics

**Reference**	**Country**	**Study Design**	**Number completed**	**Sex**	**Age (years) Mean ± SD**	**Health Status**	**Intervention**	**Polyphenol**	**Control**	**Duration** **(Weeks)**	**Outcomes**
Aboufarrag et al. [37]	United Kingdom	Crossover	52	M,F	62.6 ± 7.8	Healthy	Black rice (extract)	320 mg anthocyanin	Placebo (cellulose)	4	TC, LDL, HDL, TG, Glucose
Fauziyah et al. [50]	Indonesia	Parallel,	38	M,F	42.63 ± 7.28 (I) 43.42 ± 5.03 (C)	Healthy	Black rice (fermented, glutinous)	≥ 51.4 mg anthocyanin^a^	NR	4	LDL
Joo et al. [45]	South Korea	Parallel	48	M,F	64.96 ± 8.2 (I) 62.70 ± 6.84 (C)	Subjective memory impairment	Black rice (extract)	19.08 mg C3G	Crystalline cellulose	12	TC, LDL, HDL, TG, BP
Joo et al. [46]	South Korea	Parallel	49	M,F	44.3 ± 3.8 (I) 44.6 ± 8.3 (C)	Metabolic syndrome	Black rice (giant embryo, powdered)	46.02 mg anthocyanin^a^	White rice (powdered)	12	TC, LDL, HDL, TG, Glucose, Insulin, BMI, weight, WC
Jung et al. [47•]	South Korea	Parallel	86	F	56.91 ± 5.71 (I) 57.32 ± 5.45 (C)	Postmenopausal	Black rice (extract)	NR	Maltodextrin	12	TC, LDL, HDL, TG, Glucose, Insulin, BP, BMI, Weight
Kim et al. [48]	South Korea	Parallel	47	F	20–35 (range)	Overweight to moderately obese	Black rice (with brown rice, powder)	NR	White rice	6	TC, HDL, TG, Glucose, Insulin, BMI, weight, WC, W:H, antioxidant status
Nakamura et al. [49]	Japan	Parallel	24	NR	60.1 ± 6.3 (I) 58.0 ± 4.8 (C)	Healthy	Black rice (with brown rice)	15.2 mg anthocyanin	Polished rice	12	Postprandial Glucose, and Insulin
Seesen et al. [52]	Thailand	Parallel	62	M,F	68.80 ± 2.82 (I) 69.06 ± 2.74 (C)	Elderly	Black rice (germ powder + bran)	300 mg anthocyanins	Health education instruction	24	TC, LDL, HDL, TG, Glucose
Syarief et al. [51]	Indonesia	Parallel	52	F	42.12 ± 3.83 (I) 42.42 ± 7.49 (C)	Metabolic syndrome	Black rice (fermented, snack bar)	NR	NR	4	TC, LDL, HDL, TG
Wang et al. [53]	China	Parallel	60	M,F	63.7 ± 8.69 (I) 64.0 ± 10.7 (C)	Coronary heart disease	Black rice (pigment fraction, powder)	NR	White rice	24	TC, LDL, HDL, TG, antioxidant status

*BMI* Body Mass Index, *BP* Blood pressure, *(C)* Control, *F* female, *HDL* High density lipoprotein, *(I)* Intervention, *LDL* Low density lipoprotein, *M* male, *NR* Not reported, *RCT* randomized controlled trial, *TC* Total Cholesterol, *TG* Triglycerides, *WC* Waist circumference, *W:H* Waist-hip ratio

^a^anthocyanin based on related study

Most of the studies were from Asia, with five conducted in Thailand [38, 39, 41, 42••, 52], four in South Korea [45, 46, 47•, 48], two in Indonesia [50, 51], and one in Malaysia [43], Japan [49], and China [53], while others were from United Kingdom [37], Italy [44], and Australia [40]. Participants ranged in age from 18–75 years, were predominantly healthy [37, 38, 41, 42••, 43, 44, 49, 50], or with overweight/obesity [39, 47•, 48], Metabolic Syndrome [46, 51], CVD [53], memory impairment [45], or unspecified co-morbidities [52].

All acute studies were a cross-over design with red [40, 43], purple [39–41, 42••], and black [44] rice in various forms, including cooked [40, 42••, 43, 44], extract incorporated in sugary beverage [39] and yogurt [38], and powder used in bread [41]. These were compared against controls including rice (brown, white, basmati and jasmine), wheat bread, sucrose, and plain yogurt. All chronic studies used black rice but varied in form, such as those with giant embryo [46], in powder [48, 52, 53], extracts [45, 47•], cooked [49, 50], or incorporated into a snack bar [51, 54]. Three studies did not report a matched control [50–52]; other studies used rice (polished and white), maltodextrin, and cellulose. Duration of intervention for chronic studies was 4 [50, 51], 6 [48], 12 [45, 47•, 49], 24 [52, 53] weeks.

The polyphenol content of the pigmented rice used were reported in some studies as total polyphenols [44], flavonoids [44], anthocyanins [38, 39, 44, 49] and phenolics [43], with several including the specific content for phenolic acid (ferulic acid [49]), and anthocyanin such as cyanidin-3-glucoside [37–39, 45, 54], and peonidin-3-glucoside [37–39, 54]. In some studies, polyphenol content was derived from other studies [46, 50, 53] or not specified [40, 41, 42••, 47•, 48, 51].

### Assessment of Risk of Bias

**Fig. 2:**
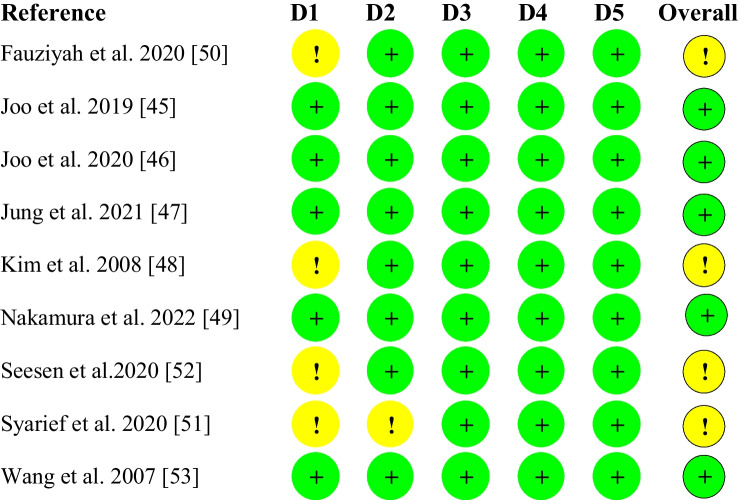
Result of the assessment for Risk of Bias of Parallel Studies. Green represents low risk of bias; yellow represents some concerns for risk of bias

Figure 2 presents the results of the risk of bias assessment for parallel studies. Four studies were assessed as having some concerns attributed to the randomization process [48, 50–52]. Despite only two studies providing a complete discussion on how randomization was carried out [45, 47•] and three providing information on blinding [45, 47•, 53], all studies provided details on baseline characteristics of participants which assisted with assessing bias arising from the randomization process (D1). All studies, except for Syarief et al. [51] provided details on the *sham* intervention provided for the control which aids in assessing bias arising from deviations from intended intervention (D2). All study outcomes are considered *observer reported outcomes not involving judgement* which are not likely to be affected by knowledge of intervention received by the participants [31].

**Fig. 3:**
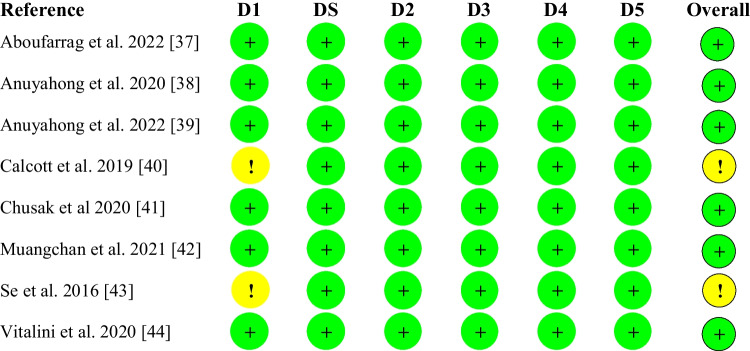
Result of the assessment for Risk of Bias for Crossover Studies. Green represents low risk of bias; yellow represents some concerns for risk of bias

Figure 3 presents the results of the risk of bias assessment for crossover studies. Two of eight studies were assessed as having some concerns as they did not provide detailed descriptions of how randomization was done. Most studies (n = 6) reported at least a one-week wash out period in between treatment groups which was considered sufficient for minimizing carry-over effects.

### Effect on Antioxidant Status

Nine studies (seven acute and two chronic) investigated the effect of pigmented rice consumption on antioxidant status [38–41, 42••, 43, 44, 48, 53]. Of the acute studies, three used Riceberry, a rice cultivar with deep-purple pigment [38, 39, 41], three provided black rice [44, 48, 53], and one study investigated both purple and red rice [40]. All acute studies reported an increase in antioxidant activity after 30 min of ingestion compared to control (brown rice, wheat bread, sucrose, or yogurt). However, peak antioxidant activity varied among studies, ranging from 30 to 360 min after ingestion (see Table 4). Duration of antioxidant activity lasted from 180 min [38, 41, 44] after ingestion, to as long as 240 [40] or 360 [39] minutes. Four acute studies demonstrated significant increases in several measures of antioxidant activity compared to control at various time points [38, 39, 41, 44], and one study reported a significant increase in FRAP compared to baseline [40].

**Table 4:** Effect of Pigmented Rice on Antioxidant Status and Postprandial Glucose and Insulin

**Reference** **(sample size)**	**Antioxidant Status**	**Glucose**	**Insulin**
**Acute Studies**			
Anuyahong et al. [38], (*n* = 19)	• Significantly increased compared to control at • **60**, 90 and 120 min (FRAP) • 30, **90**, 120 and 150 min (TEAC) • 30, 60, 90 and **120** min (ORAC) • No significant difference in Thiol compared to control at all time points • Significantly lower MDA at 60, 90, and 180 min compared to control	• Significantly lower compared to control at 30 and 120 min	N/A
Anuyahong et al. [39], (*n* = 13)	• Significantly increased compared to control at • **120**, 180, 240, and 360 min (FRAP) • 30, 90, **240**, and 300 min (TEAC) • 60, 90, 180, 240, 300, **360** min (Thiol)	• Significantly lower compared to control at 30, 60, 90 and 120 min	• Significantly lower compared to control at 60 and 90 min
Callcott et al. [40], (*n* = 22)	• Significantly increased FRAP from baseline at **60** min for purple rice; and at **30** and 60 min for red rice. No significant increase from baseline for control • Significantly reduced MDA from baseline at 30 min for purple rice and 240 min for red rice. No significant reduction from the baseline for control	N/A	N/A
Chusak et al. [41], (*n* = 16)	• Significantly increased FRAP compared to control at 120 and **150** min • No significant difference in Thiol compared to control • No significant difference in MDA compared to control	• Significantly lower compared to control at 30 and 60 min	• Significantly lower compared to control at 15 and 60 min
Muangchan et al. [42••], (*n* = 6)	N/A	• Significantly lower compared to control at 60 min	• No significant difference compared to the control
Se et al. [43], (*n* = 12)	N/A	• No significant difference between groups at any timepoints	• No significant difference compared to control
Vitalini et al. [44], (*n* = 19)	• Significantly increased DPPH and ABTS compared to control at **60** and 120 min	N/A	N/A
**Chronic Studies**			
Kim et al. [48], (*n* = 47)	• Change in SOD after six months was not significant between groups	N/A	N/A
Nakamura et al. [49], (*n* = 24)	N/A	• No significant difference between groups at any timepoints	• Significantly lower insulin at 120 min compared to control
Wang et al. [53], (*n* = 60)	• Change in FRAP after six months significantly different between groups • Change in SOD after six months was not significant between groups	N/A	N/A

Timepoints of peak antioxidant activity are in **bold**

*ABTS* 2,20-azino-bis(3-ethylbenzothiazoline-6-sulphonic acid, *DPPH* 2,2-diphenyl-1-picrylhydrazyl, *F* female, *FRAP* ferric reducing ability of plasma, *MDA* Malondialdehyde, *N/A not applicable*, *ORAC* oxygen radical absorbance capacity, *SOD* superoxide dismutase, *TEAC* Trolox equivalent antioxidant capacity

Wang et al. [53] measured antioxidant activity after six months of intervention with black rice fraction among CHD patients. They demonstrated significantly higher FRAP activity compared to control (1.29 ± 2.96 103 u/L vs. -0.61 ± 1.69 103 u/L; p < 0.01) but no differences in superoxide dismutase (SOD). Similarly, Kim et al. [48] also showed no effect on SOD, but observed significantly higher glutathione peroxidase among women with obesity who consumed black rice meal replacements for six weeks compared to white rice meal replacements (15.36 ± 5.63 U/g Hb vs. 3.52 ± 5.41 U/g Hb, p < 0.05).

### Effect on Lipids

**Fig. 4:**
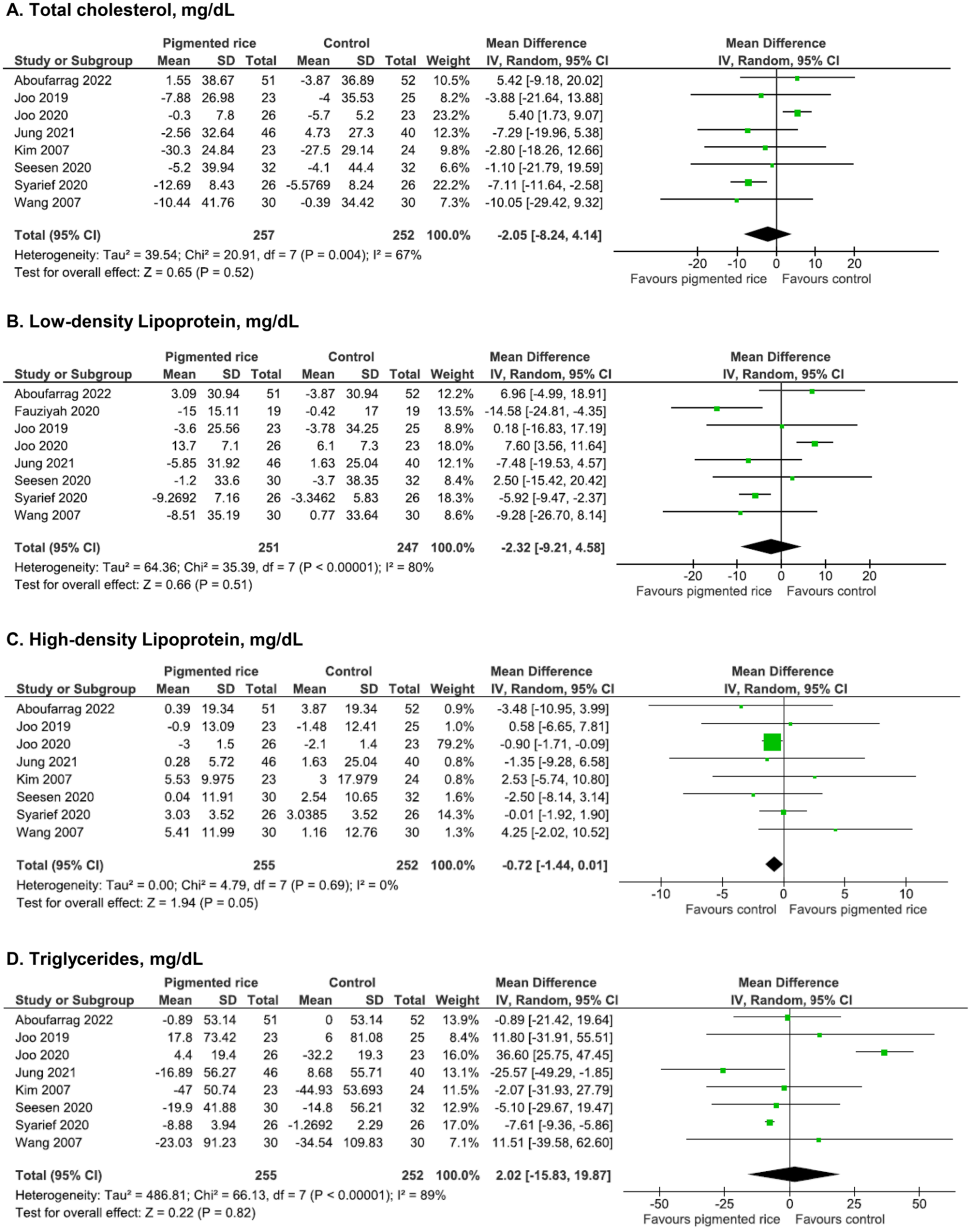
Effect of pigmented rice on lipid profile

All studies (n = 8, all chronic) that evaluated changes in TC*,* LDL, HDL*,* and TG were included in the meta-analysis. Significant heterogeneity can be observed among the pooled studies for TC (*I*^*2*^ = 67%), LDL (*I*^*2*^ = 80%), and TG (*I*^*2*^ = 89%). Pigmented rice consumption did not improve TC (WMD -2.05 mg/dL; 95% CI:-8.24,4.14, p = 0.52, *n* = 509, Fig. 4a), LDL (WMD -2.32 mg/dL; 95% CI:-9.21,4.58, *n* = 498, p = 0.51, Fig. 4b), HDL (WMD = -0.72 mg/dL; 95% CI:-1.44,0.01, p = 0.05, *n* = 507, Fig. 4c), or TG (WMD = 2.02 mg/dL; 95% CI:-15.83,19.87, p = 0.82, *n* = 507, Fig. 4d) compared to control.

### Effect on Glucose

Eleven studies reported glucose as an outcome (five acute and six chronic intake). All data for postprandial glucose and insulin were reported in line graphs, which limited data extraction (only one of the contacted authors provided data). Only the chronic studies provided sufficient data for meta-analysis.

**Fig. 5:**
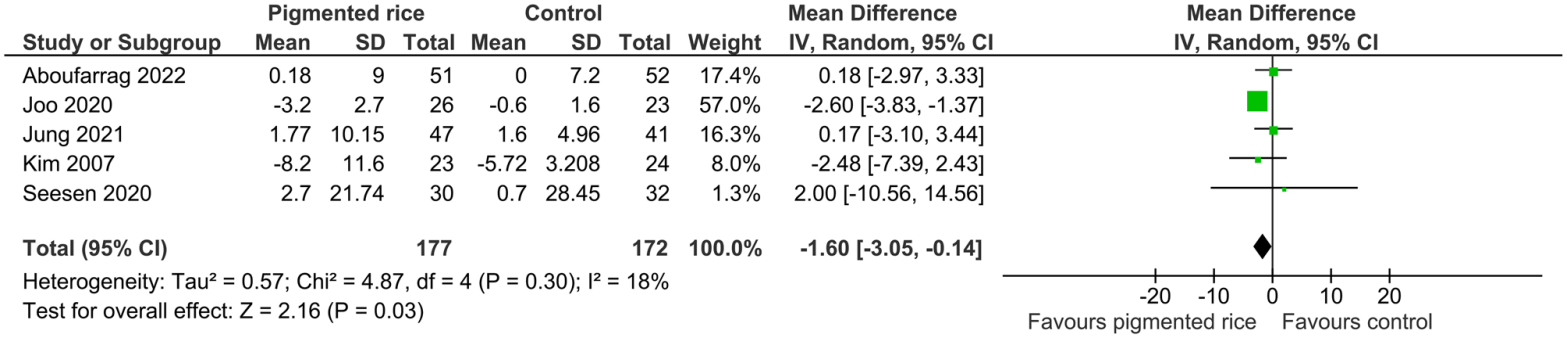
Effect of pigmented rice on glucose

The majority of acute studies reported significantly lower postprandial glucose levels compared to control (plain yogurt, sucrose, wheat bread, white rice) [38, 39, 41, 42••] starting at 30 min up to 120 min after ingestion (Table 4). Additionally, three out of five studies that measured postprandial insulin reported significantly lower insulin levels compared to their control (sucrose, wheat bread, polished rice) [39, 41, 49] ranging from 15 min up to 120 min after ingestion (Table 4). Compared to control, chronic pigmented rice consumption significantly lowered fasting glucose (WMD = -1.60 mg/dL; 95% CI: -3.05, -0.14, *I2* = 18%, p = 0.03, *n* = 349, Fig. 5).

### Effect on Blood Pressure

**Fig. 6:**
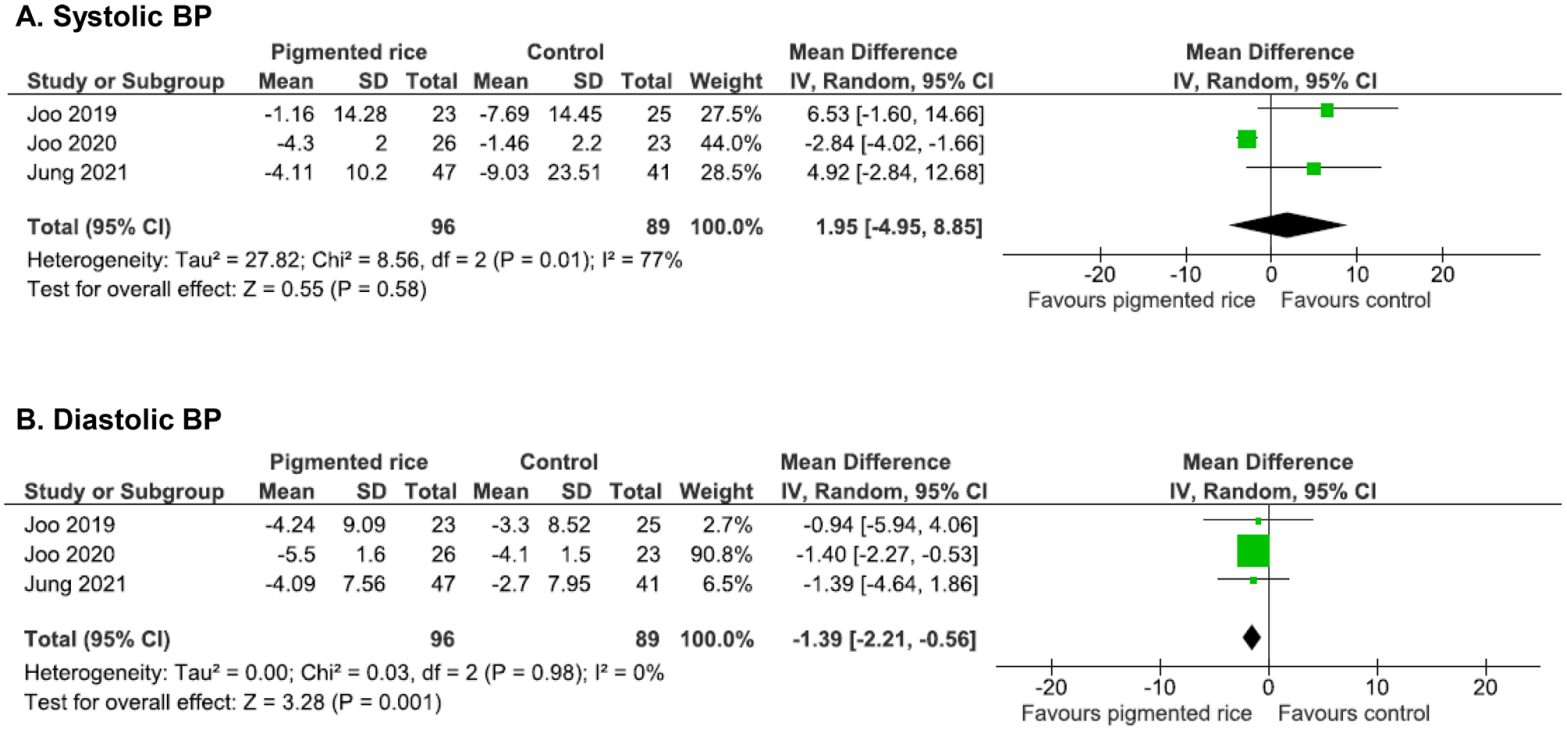
Effect of pigmented rice on blood pressure

Three chronic studies included BP as an outcome [45, 46, 47•]. Significant improvements were observed for diastolic BP only (WMD = -1.39 mmHg, 95% CI: -2.21, -0.56, I2 = 0%, p = 0.001, *n* = 185, Fig. 6b).

### Effect on Weight and Body Mass Index

**Fig. 7:**
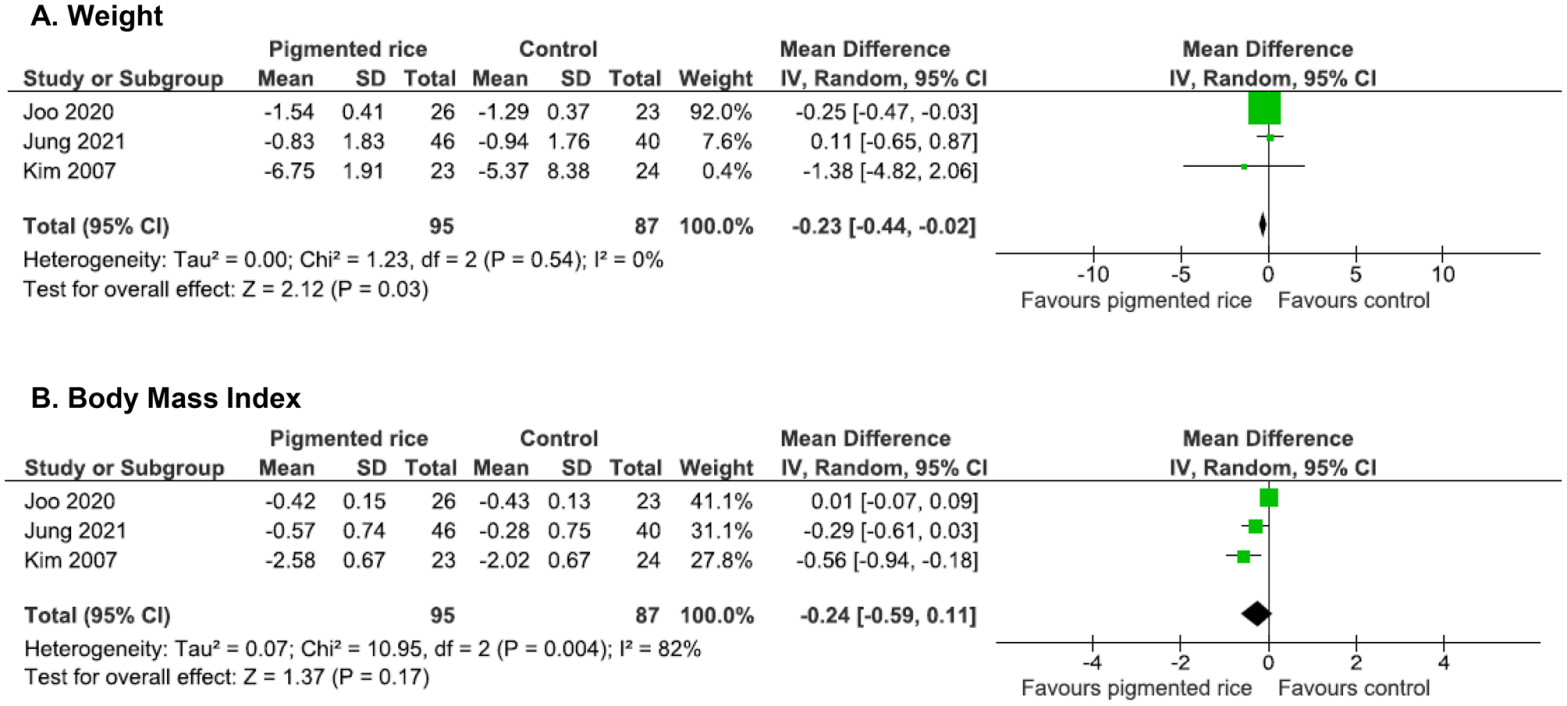
Effect of pigmented rice on weight and Body Mass Index

Three chronic studies included weight and BMI as outcomes [46, 47•, 48]. Pigmented rice consumption was associated with significant reductions in weight (WMD = -0.23 kg, 95% CI: -0.44, -0.02, *I*^*2*^ = 0%, p = 0.03, *n* = 182, Fig. 7a), but not BMI (WMD = -0.24 kg/m^2^, 95% CI: -0.59, 0.11, *I*^*2*^ = 82, p = 0.17, *n* = 182, Fig. 7b).

### GRADE

The certainty assessment for each outcome is presented in Table 5. All outcomes were assessed to have no serious risk of bias. However, TC, LDL, TG, Glucose, and SBP have serious concerns for inconsistency and indirectness attributed to differences in the study population, and delivery of the intervention. Lastly, imprecision was assessed as serious for TC, LDL, BMI, weight, and DBP, and very serious for HDL, TG, and SBP.

**Table 5:** GRADE Assessment for Certainty of Evidence

Sample size (studies)	Risk of bias	Inconsistency	Indirectness	Imprecision	Publication bias	Overall certainty of evidence
Total Cholesterol						
509 (8 RCTs)	not serious	serious^a,b,c^	serious^a^	serious^d^	none	⨁ ○ ○ ○ Very Low
Low Density Lipoprotein						
498 (8 RCTs)	not serious	serious^a,b,c^	serious^a^	serious^d^	none	⨁ ○ ○ ○ Very Low
High Density Lipoprotein						
507 (8 RCTs)	not serious	not serious	not serious	very serious^e^	none	⨁ ⨁ ○ ○ Low
Triglycerides						
507 (8 RCTs)	not serious	very serious^a,b,c^	very serious^a^	very serious^d^	none	⨁ ○ ○ ○ Very Low
Glucose						
349 (5 RCTs)	not serious	not serious	very serious^a^	not serious	none	⨁ ⨁ ○ ○ Low
Systolic Blood Pressure						
185 (3 RCTs)	not serious	serious^a,b,c^	not serious	very serious^d^	none	⨁ ○ ○ ○ Very Low
Diastolic Blood Pressure						
185 (3 RCTs)	not serious	not serious	serious^a,c^	serious^e^	none	⨁ ⨁ ○ ○ Low
Weight						
182 (3 RCTs)	not serious	not serious	not serious	serious^e^	none	⨁ ⨁ ⨁ ○ Moderate
Body Mass Index						
182 (3 RCTs)	not serious	very serious^a,b,c^	not serious	serious^d^	none	⨁ ○ ○ ○ Very Low

^a^Differences in study population

^b^Substantial heterogeneity

^c^Differences in intervention (form, dosage, and duration)

^d^Overall confidence interval crosses the threshold

^e^Confidence interval of included studies crossed the threshold

## Discussion

This study systematically reviewed the effect of pigmented rice consumption on antioxidant status and cardiometabolic risk factors in adults. Meta-analysis of chronic intake studies demonstrated significant reductions in glucose, weight, and diastolic BP, but no significant effects on TC, LDL, TG, HDL, BMI, or systolic BP. All acute studies included in the review demonstrated that pigmented rice consumption increases antioxidant activity 30 min after ingestion and is sustained for at least 180 min. Moreover, chronic consumption of pigmented rice (12–24 weeks) was shown to increase total antioxidant activity compared to control [48, 53]. This finding may explain the role of pigmented rice in improving blood pressure by reducing oxidative stress which promotes endothelial dysfunction [55]. Reactive oxygen species decrease nitric oxide production and promote cellular damage, resulting to inflammation, vasoconstriction, vascular lesion and ultimately atherosclerosis and CVDs [56].

The antioxidant activity of pigmented rice is attributed to its polyphenol content such as phenolic acids that can stabilize electrons, and flavonoids (including anthocyanins) that have the ability to donate electrons and stop chain reactions [20]. Various studies have shown pigmented rice promotes radical scavenging activity (DPPH) [44], has reducing power (FRAP) [38, 39, 41, 53], ability to inhibit radical induced oxidation (ORAC [38] and ABTS [44]), ability to neutralize radical cation (TEAC) [38–40], and reduces oxidative stress (MDA) [38–41]. Despite having low bioavailability, once absorbed in the gut anthocyanins are metabolized in the liver, secreted and reabsorbed in the enterohepatic circulation resulting in molecular intermediates that contribute to their biologic actions [57].

The meta-analysis showed significant beneficial effects on glucose, weight, and diastolic BP, but not cholesterol (TC, LDL, HDL, TG), BMI, or systolic BP. Black rice anthocyanins may help reduce glucose levels by delaying carbohydrate absorption through inhibition of α-amylase and α-glucosidase [58]. This can be seen in the acute intake studies where the majority demonstrated significantly lower postprandial glucose and insulin levels at various timepoints compared to the control. This may imply that pigmented rice consumption may help maintain lower blood glucose levels post meal. However, it should be noted that despite significant reductions in fasting blood glucose levels in chronic studies, the magnitude of effect was small and unlikely to be considered clinically meaningful. Participants in this analysis had normal blood glucose levels at baseline; it is possible that greater benefit may be seen in persons with elevated glucose levels. Additionally, polyphenols may assist with managing obesity through increased energy expenditure, appetite suppression, and regulation of lipid metabolism [59]. Several mechanisms of action have been postulated for how pigmented rice consumption may reduce lipid levels. These include regulation of fatty acid synthesis [25, 26], transcription factors [29], and lipid metabolism [26]. However, while most studies included in this review reported reductions in lipids, these were not statistically different to the control, and collectively this meta-analysis did not show any benefit on lipids. Manach et al. [60] argues that doses in animal studies are often higher than what human tissue may be exposed to. Additionally, participants in many of the studies included in this review had normal TC, LDL, TG, and glucose levels, as opposed to most animal studies which induce hyperlipidemia or hyperglycemia.

Moreover, considerable differences in study characteristics were noted. While all studies in the meta-analysis used black rice, they varied in form (cooked, extract, fermented, glutinous) and dosage (15 mg to 320 mg anthocyanin). Phenolic acids, mostly ferulic acid, and anthocyanins which are mostly cyanidin 3-*O*-glucodside and peonidin 3-*O*- glucoside, are highly concentrated in the bran [20]. Further, different rice forms (extract, with giant embryo, glutinous) and effect of processing (cooked, fermented, incorporated to a snack bar) may contribute to differences in concentration and bioavailability of polyphenols. Black sticky rice was reported to have higher antioxidant activity compared with red, and black rice [19•]. Germination of rice also increase antioxidant activity, total phenolics and flavonoids [61], while fermentation of grains is hypothesized to increase bioavailability of phenolic compounds [62]. The study of Fauziyah et al. [50] which utilized fermented glutinous black rice reported the greatest reduction in LDL among all studies included in this review, which was significantly lower compared to the control. Most of the chronic studies matched their control with the intervention, either using white/polished rice in the same form as the intervention (i.e., whole, powdered, meal replacement mix) or using placebo/maltodextrin capsules identical to the extracts administered to the intervention group. However, some studies did not report the specific control used [50, 51], as noted in the assessment of risk of bias, or did not attempt to match the rice form given to the intervention group [52]. Lastly, included studies differed in duration of intervention, and presence or absence of co-intervention. Apart from the rice, one study also provided diet counselling to both groups [50]; one study induced energy restriction in both groups [48]. Seesen et al. [52], provided health education to the control in lieu of the intervention. Due to the limited number of studies, subgroup analyses could not be conducted to determine whether these differences impacted on the cardiometabolic outcomes. Altogether, these differences influence the certainty of evidence of the outcomes. Therefore, further randomized controlled human clinical trials are warranted to support the clinical value of pigmented rice consumption for reducing cardiometabolic risk factors.

The present meta-analysis has some limitations that should be noted. First, some of the included studies have some concerns based on Cochrane Collaboration’s Risk of Bias, specifically in relation to how randomization was carried out. Secondly, no meta-analysis was conducted on antioxidant status, or postprandial glucose and insulin due to availability of data from included studies. Outcomes with meta-analyses have heterogeneity (TC, LDL, TG, BMI, and systolic BP) which is likely attributed to differences in study design.

## Conclusion

This systematic review showed that acute intake of pigmented rice increases antioxidant activity and lowers postprandial glucose and insulin levels. Meta-analysis demonstrated significant reductions in glucose, weight, and diastolic BP following chronic pigmented rice consumption, but no significant effects on TC, LDL, TG, HDL, BMI, or systolic BP. More high quality randomized controlled trials are warranted to further investigate the effect of pigmented rice consumption on cardiometabolic risk factors in adults; additional benefit may be observed in those with established clinical conditions such as dyslipidemia, pre-diabetes/diabetes, and hypertension.

### Supplementary Information

Below is the link to the electronic supplementary material.

Supplementary file1 (DOCX 14 KB)

## Data Availability

The datasets used and/or analysed during the current study are available from the corresponding author on reasonable request.
