# Supplementary material for: Effect of Pigmented Rice Consumption on Cardiometabolic Risk Factors: A Systematic Review of Randomized Controlled Trials
Source: Curr Nutr Rep. 2023 Sep 7;12(4):797–812. doi: 10.1007/s13668-023-00496-7 (PMC10766681; doi:10.1007/s13668-023-00496-7)
Supplement: Supplementary file 1 — Supplementary file1 (DOCX 14 KB) [file 13668_2023_496_MOESM1_ESM.docx]

**Supplemental Table s1** Search Strategy

| **Category** | **Search concepts** |
| --- | --- |
| Population | Adult [Mesh] OR adult OR "Healthy Volunteers"[Mesh] OR subject |
| Intervention | Oryza[Mesh] OR oryza OR "pigmented rice" OR "colored rice" OR "pigment rice" OR "red rice" OR "black rice" OR "purple rice" OR "violet rice" OR "oryza sativa" OR Flavonoid* OR Anthocyanin* OR "Phenolic acid" OR "Proanthocyanidin rice" |
| Comparator | "non pigmented rice" OR "white rice" OR "brown rice" OR maltodextrin OR "usual diet" OR placebo* OR control OR randomi* |
| Outcomes | Cholesterol OR "Cholesterol, HDL"[Mesh] OR "Cholesterol, LDL"[Mesh] OR "Triglycerides"[Mesh] OR lipids OR “lipids”[Mesh] OR “Blood Glucose”[Mesh] OR “Blood Glucose” OR “Fasting blood glucose” OR Insulin OR “insulin sensitivity” OR "Glycated Hemoglobin A"[Mesh] OR "Glycated Hemoglobin A" OR "Glycemic Control"[Mesh] OR "Glycemic Control" OR “antioxidant status” OR “Oxygen Radical Absorbance Capacity”[Mesh] OR “Oxygen Radical Absorbance Capacity” OR “radical scavenging assay” OR “radical scavenging” OR “ferric reducing antioxidant power“ OR ”Fluorescence Recovery After Photobleaching” OR "1,1-diphenyl-2-picrylhydrazyl" OR "2,2'-azino-di-(3-ethylbenzothiazoline)-6-sulfonic acid" OR “Total antioxidant capacity” OR “Total radical activity” OR antiradical OR “Total phenol index” OR ”antioxidant capacity” OR “body weight”[Mesh] OR "body weight" OR "Body Mass Index"[Mesh] OR "Body Mass Index" OR "Obesity"[Mesh] OR obes* OR overweight[Mesh] OR overweight OR "Metabolic Syndrome"[Mesh] OR “metabolic syndrome” OR "Waist Circumference"[Mesh] OR "Waist Circumference" OR "Waist-Hip Ratio"[Mesh] OR "Waist-Hip Ratio" OR “blood pressure” OR “diastolic blood pressure” OR diastolic OR “systolic blood pressure” OR systolic |
